# Malaria in Eswatini, 2012–2019: a case study of the elimination effort

**DOI:** 10.1186/s12936-021-03699-x

**Published:** 2021-03-20

**Authors:** Theresia Estomih Nkya, Ulrike Fillinger, Makhoselive Dlamini, Onyango P. Sangoro, Rose Marubu, Zulisile Zulu, Emmanuel Chanda, Clifford Maina Mutero, Quinton Dlamini

**Affiliations:** 1 International Centre of Insect Physiology and Ecology, Nairobi, Kenya; 2 World Health Organization, Eswatini Country Office, Mbabane, Eswatini; 3 National Malaria Programme, Ministry of Health, Mbabane, Eswatini; 4 World Health Organization, Regional Office for Africa, Brazzaville, Congo; 5 University of Dar Es Salaam, Mbeya College of Health and Allied Sciences, Mbeya, Tanzania; 6 University of Pretoria Institute for Sustainable Malaria Control, Pretoria, South Africa

**Keywords:** Malaria, Surveillance, Elimination, Integrated vector management

## Abstract

Eswatini was the first country in sub-Saharan Africa to pass a National Malaria Elimination Policy in 2011, and later set a target for elimination by the year 2020. This case study aimed to review the malaria surveillance data of Eswatini collected over 8 years between 2012 and 2019 to evaluate the country’s efforts that targeted malaria elimination by 2020. Coverage of indoor residual spraying (IRS) for vector control and data on malaria cases were provided by the National Malaria Programme (NMP) of Eswatini. The data included all cases treated for malaria in all health facilities. The data was analysed descriptively. Over the 8 years, a total of 5511 patients reported to the health facilities with malaria symptoms. The case investigation rate through the routine surveillance system increased from 50% in 2012 to 84% in 2019. Incidence per 1000 population at risk fluctuated over the years, but in general increased from 0.70 in 2012 to 1.65 in 2019, with the highest incidence of 3.19 reported in 2017. IRS data showed inconsistency in spraying over the 8 years. Most of the cases were diagnosed by rapid diagnostic test (RDT) kits in government (87.6%), mission (89.1%), private (87%) and company/industry-owned facilities (84.3%), either singly or in combination with microscopy. Eswatini has fallen short of achieving malaria elimination by 2020. Malaria cases are still consistently reported, albeit at low rates, with occasional localized outbreaks. To achieve elimination, it is critical to optimize timely and well-targeted IRS and to consider rational expansion of tools for an integrated malaria control approach in Eswatini by including tools such as larval source management, long-lasting insecticidal nets (LLINs), screening of mosquito house entry points, and chemoprophylaxis. The establishment of rigorous routine entomological surveillance should also be prioritized to determine the local malaria vectors’ ecology, potential species diversity, the role of secondary vectors and insecticide resistance.

## Background

Globally, more countries are moving towards zero indigenous malaria cases. In 2018, 49 countries reported fewer than 10,000 malaria cases [1]. The number of countries with fewer than 100 indigenous cases increased from 17 in 2010 to 25 in 2017 and 27 in 2018 [1]. In 2016, the World Health Organization (WHO) identified 21 countries with the potential to eliminate malaria by 2020, the E-2020 initiative, and resolved to work with their governments to support their elimination goals [2]. Eswatini is among the E-2020 countries and part of the Elimination 8 (E-8), a regional initiative established in 2009 by the Southern African Development Community (SADC). The E-8 initiative is coordinating a collaborative effort, led by the ministers of health in eight countries (Botswana, Namibia, South Africa, Eswatini, Angola, Mozambique, Zambia, and Zimbabwe) to jointly plan and execute a regional malaria elimination strategy. The E-8 aims to mitigate cross-border transmission, which presents a major threat to the re-establishment of infection [3].

**Fig. 1:**
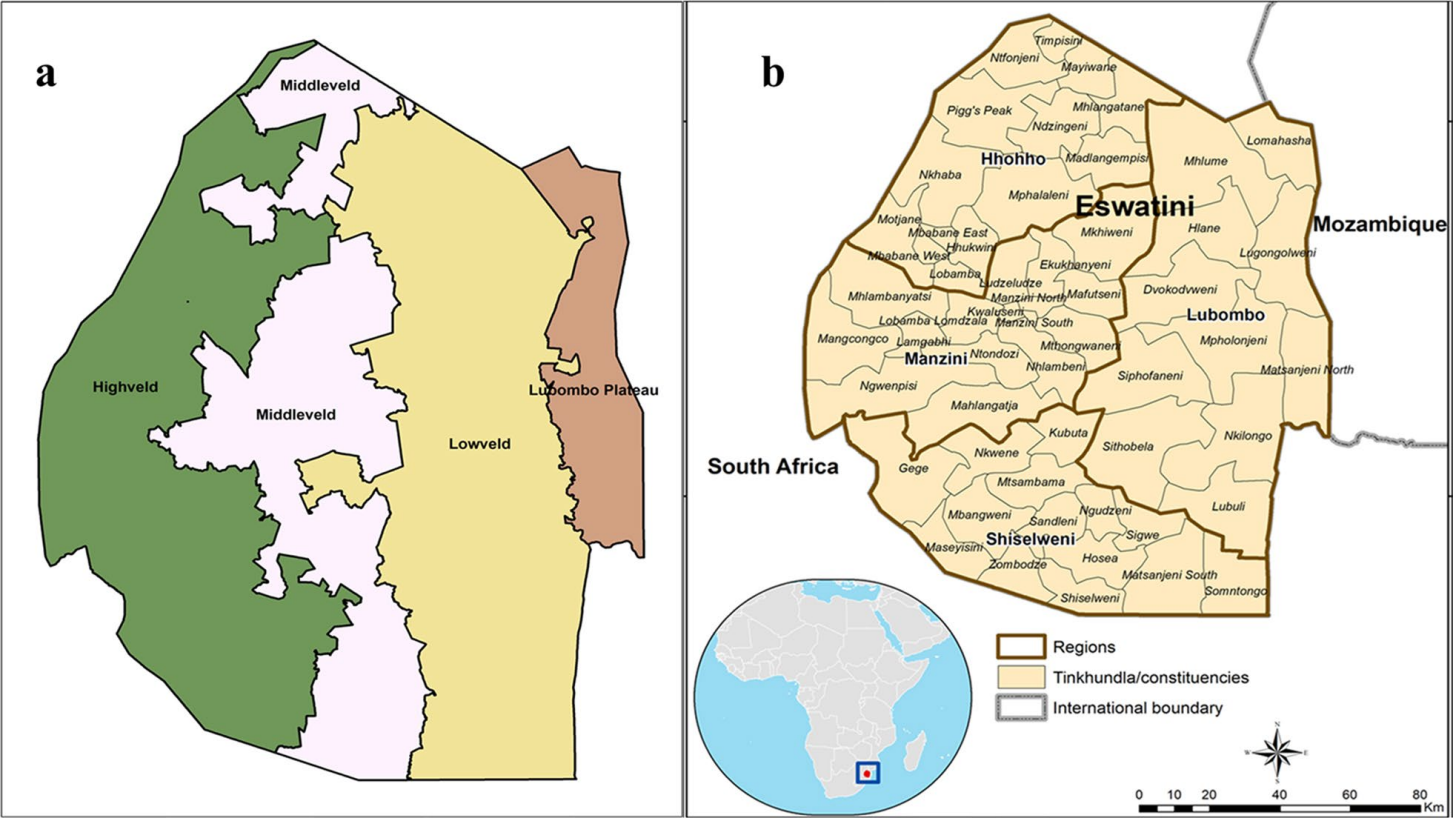
Map of Eswatini is a landlocked country surrounded by South Africa and Mozambique. **a** Ecological regions; highveld, middleveld, and lowveld. **b** Eswatini showing regions, constituencies, and international borders

In Eswatini, malaria transmission is seasonal and highly influenced by variations in altitude through the corresponding effects of rainfall and temperature levels. The country is divided into four ecological regions distinguished by elevation, climate, soil quality and vegetation: highveld (altitude above 1500 m); middleveld (average altitude 700 m); and, lowveld (average elevation 400 m) [4] (Fig. 1a). Historically, malaria transmission has been confined in the lowveld and lower areas of middleveld regions, where malaria vector breeding is favoured by a range of environmental factors, including warm and wet autumn and summer seasons, and availability of suitable mosquito breeding habitats. Before the commencement of vector control measures in 1949, malaria was a major health problem in Eswatini, with epidemics reported during the summer and autumn months from December to May [4]. Malaria control measures were extremely limited and prejudicial, whereby the Europeans living in the lowveld regions were advised to put screens on their windows and to avoid walking outdoors in the evenings, while no similar health instructions were given to the native Swazi. In 1946, during the first epidemic, where extensive malaria surveys were carried out, it was estimated that 50,000 cases occurred, which corresponded to 26% of the total population of Eswatini at the time [5]. These epidemics were attributed to heavy rainfall which led to an increase in vector breeding sites and colonial economic policies, which prevented many Swazi families from producing enough food to meet their subsistence needs [4]. Following the successful control of malaria with indoor residual spraying (IRS) using dichloro-diphenyl-trichloroethane (DDT) in the 1950s and early 1960s in the lowveld, agricultural activities could now be intensified in these regions. This led to the construction of major irrigation schemes for sugar plantations which, unfortunately, resulted in a resurgence of malaria in the areas in which sugar was grown, undermining the effectiveness of the malaria control measures that had been put in place. Autochthonous cases of malaria occurred around the sugar estates in 1960 and larger outbreaks followed in 1967 and 1972 [5]. The number of recorded cases continued to rise during the late 1970s and began to spread out from the sugar estates to other areas of the lowveld and into the lower parts of the middleveld. Ineffective malaria control measures within the sugar estates, and more widely in the lowveld, led to the creation of ideal breeding sites for malaria vectors within the irrigation projects. Demographic shifts, with more non-immune populations living near malaria vectors and carriers, further contributed to the re-establishment of malaria as a serious health problem in Eswatini in the late 1970s.

However, between 1999 and 2009, Eswatini scaled up vector control, largely using IRS in the at-risk regions and border areas and established a cross-border collaboration with Mozambique and South Africa for malaria control [6]. As a result, Eswatini greatly reduced the national burden of malaria from 3.9 laboratory-confirmed cases to 0.07 cases per 1000 population [7]. The successful control of malaria through national and cross-border efforts positioned Eswatini to be earmarked for elimination by 2015 by the SADC and the African Union [8, 9] and the National Strategic Plan for Elimination (NMESP) of Malaria in Eswatini was initiated [7]. The NMESP for 2008–2015 set the country on a malaria elimination path. In March 2011, Eswatini became the first country in sub-Saharan Africa to approve a National Malaria Elimination Policy [7]. As defined in the NMESP 2015–2020, Eswatini’s plan to eliminate malaria focused on four major intervention areas: case management; vector control with IRS; surveillance; and information, education, and communication on malaria [10]. With the introduction of rapid diagnostic test (RDT) kits at all health facilities in February 2010, laboratory-confirmed cases increased marginally while the number of clinically diagnosed cases decreased significantly, indicating successful uptake of RDT use [11]. Additionally, a surveillance programme has been operationalized nationally to facilitate the investigation of confirmed malaria cases at the household level to determine the source of each infection. Community-based case detection was established to help identify asymptomatic infections that contribute to ongoing local transmission. This has allowed the identification of high-risk groups and areas that can be targeted with additional interventions, including vector control using IRS and health promotion messages [10, 11]. At the core of Eswatini’s National Malaria Programme (NMP) vector control strategy is IRS targeted at areas of high malaria transmission/burden. IRS guidelines direct that the entire populations living in those areas have all rooms of their houses sprayed once a year prior to the malaria season. Furthermore, in response to each confirmed local case, and in the event of local malaria epidemic, additional spatially targeted IRS campaigns are to be implemented alongside vector surveillance. However, in recent years, IRS activities have been scaled down to a more targeted approach (as opposed to blanket spraying) in malaria hotspots [10].

This case study aimed to review the malaria surveillance data of Eswatini collected over 8 years between 2012 and 2019 to evaluate the country’s efforts that targeted malaria elimination by 2020.

## Methods

### Study setting

Eswatini is a landlocked country in the southern part of Africa bordered by South Africa and Mozambique (Fig. 1b). Malaria transmission is seasonal in Eswatini, due to the country’s subtropical climate, and occurs during the warmer and wetter months of November to April. From May to October, it is cooler and drier (winter) and malaria transmission normally ceases, except for a few malaria hotspots in the riverine areas of the lowlands [12]. Of the 1,172,433 population, an estimated 30% live in communities that are prone to malaria transmission (Table 1) [13]. *Plasmodium falciparum*, is responsible for > 99% of malaria cases, while the main vector is reported as *Anopheles arabiensis* [14], even though there is a scarcity of up-to-date entomological data to support this assertion [3]. Eswatini’s mobile population and labour force contribute to sustaining the malaria risk in the country, especially across the border with Mozambique, where malaria remains a major public health issue. According to the Service Availability and Readiness Assessment (SARA) of 2017, Eswatini had 327 health facilities [15] providing services to most households within an 8 km radius. Facility ownership was distributed between government-owned facilities (39%), facilities privately owned by doctors or nurses (29%), mission-owned facilities (13%), industry-owned facilities (10%), and non-governmental-organization-owned facilities (9%).

**Table 1:** Population at risk of malaria in Eswatini, 2012–2019

Year	Total population^a^	Population at risk (30%)
2012	1,080,337	324,101
2013	1,093,158	327,947
2014	1,106,189	331,857
2015	1,119,375	335,813
2016	1,132,657	339,797
2017	1,145,970	343,791
2018	1,159,250	347,775
2019	1,172,433	351,730

^a^Total populations are projections from Eswatini population projections, 2007*–*2030 [13]

The NMESP for 2008–2015 led to the revision of the country's diagnostic and treatment guidelines and the adoption of the WHO guidelines for low-transmission settings [16, 17]. The revised guidelines required that all cases of fever be confirmed for malaria infection by RDT or microscopy before treatment was initiated. Artemether–lumefantrine (AL) was the drug of choice for uncomplicated cases, and quinine for severe cases and as first-line treatment for pregnant women in their first trimester of pregnancy [18]. The guidelines underwent revision in 2014 and 2017. The latest National Malaria Diagnosis and Treatment Guidelines replaced parenteral quinine with parenteral artesunate as the first-line treatment for severe and complicated malaria and single, low-dose primaquine (0.25 mg/kg) in addition to AL are used for the treatment of uncomplicated *P. falciparum* malaria [19].

### Eswatini’s malaria case surveillance

**Fig. 2:**
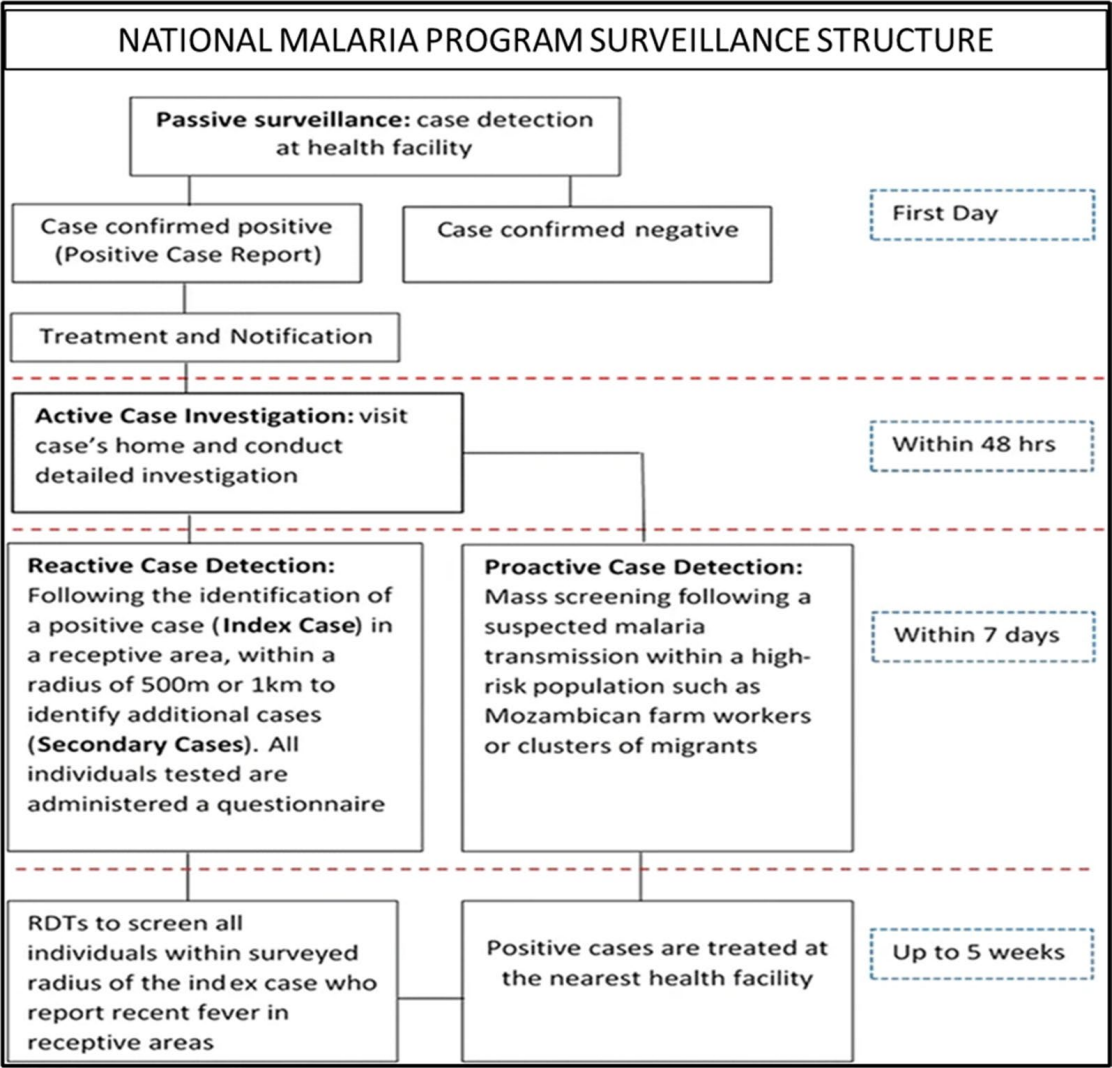
Eswatini’s national malaria program surveillance structure

Case notification is through the Instant Disease Notification System (IDNS) hosted by Emergency Preparedness and Response (EPR) for notification of diseases reported from health facilities by call and the IDNS sends SMS to the NMP surveillance for a response. This system allows the health care worker to capture demographic details about the patient that assist in patient follow-up. The NMP carries out active surveillance which involves; active case investigation in the household of the index case, triggered by parasitological confirmation of a malaria case at a health facility; reactive case detection (RACD), triggered by the location of a confirmed malaria case in Eswatini’s receptive area; and, pro-active case detection, triggered by a strong suspicion of malaria transmission within a defined detection area and on high-risk populations. The index case, whether it is identified by RDT and/or by microscopy, is investigated at the patient’s home within 48 h of the patient’s presentation date, subject to consent by the patient or guardian. The case investigation’s primary purpose is to establish the case origin (imported or autochthonous) and collect other relevant demographic data such as global positioning system (GPS) coordinates, treatment received, age, gender, nationality, and occupation of the patient. If the confirmed malaria case lives in a receptive area, every person residing within a radius of either 1 km or 500 m from the residence of the index case is tested for malaria. A RACD event remains open for up to 5 weeks, where the NMP additionally conducts fever screening and where individuals near the index case report a recent fever, enabling identification of additional secondary cases. Any identified positive case is referred to the nearest health facility for treatment and followed up. The active surveillance programme is implemented in all regions of the country, however; RACD only takes place in receptive areas, determined by mapping the locations of historic cases and vector surveillance data (Fig. 2).

### Review of data

This was a descriptive retrospective study utilizing data routinely collected using IDNS from the health facilities and reported to the Eswatini NMP between 2012 and 2019. The data included cases treated for malaria in all health facilities of Eswatini reported to NMP and entered in the active case investigation database; including confirmed cases (RDT and/or microscopy), investigated cases (followed up at household level), case origin (autochthonous and imported cases), and demographic data (nationality, age, and gender). The terminology used is as per WHO definitions (Table 2).

**Table 2:** Definitions of terminology used based on WHO [20, 21]

Type of malaria	Description
Surveillance (elimination programmes)	That part of the programme designed for the identification, investigation and elimination of continuing transmission, the prevention and cure of infections, and final substantiation of claimed elimination
Confirmed case	A malaria case or infection in which the parasite has been detected in a diagnostic test, i.e., microscopy, a rapid diagnostic test, or a molecular diagnostic test
Presumed case	A malaria case suspected of being malaria is not confirmed by a diagnostic test
Active case detection	Detection by health workers of malaria cases at community and household levels, sometimes in population groups that are considered at high risk
Passive case detection	Detection of malaria cases among patients who, on their initiative, visit health services for diagnosis and treatment, usually for a febrile illness case
Confirmed case	Malaria case (or infection) in which the parasite has been detected in a diagnostic test, i.e., microscopy, a rapid diagnostic test, or a molecular diagnostic test
Indigenous case	A case contracted locally with no evidence of importation and no direct link to transmission from an imported case
Introduced case	A case contracted locally, with strong epidemiological evidence linking it directly to a known imported case (first-generation local transmission)
Imported case	Malaria case or infection in which the infection was acquired outside the area in which it is diagnosed
Autochthonous	A case locally acquired by mosquito-borne transmission, i.e., indigenous or introduced case (also called ‘locally transmitted’)

The IRS data for the same period was also reviewed. According to national guidelines, IRS is supposed to be carried out annually in October (one spray cycle) in malaria-endemic areas. Over the study period, insecticides used for IRS were DDT, lambda-cyhalothrin and pirimiphos-methyl. DDT was sprayed in mud structures and lambda-cyhalothrin in modern/cement structures. Spray coverage was obtained from the NMP records and was based on the number of structures reported to have been sprayed between 2014 and 2019. Lack of data for 2012–2013, as reported by NMP, was due to a technical malfunction of their servers that led to the loss of data records.

### Data analysis

The study variables included case status (investigated or not investigated), case origin (autochthonous, imported), demographics of patients (age, gender, and nationality), IRS coverage (coordinates of sprayed structures), health facility (government, mission/NGO and private) and method of diagnosis (RDT and microscopy) as well as treatment. Data were entered into Microsoft Office Excel 2010 (Microsoft Corp., Redmond, WA) and SPSS 19.0 software (IBM) for analysis. The incidence rate was calculated from the confirmed number of cases per 1000 population at risk for each year (Table 1).

## Results

**Fig. 3:**
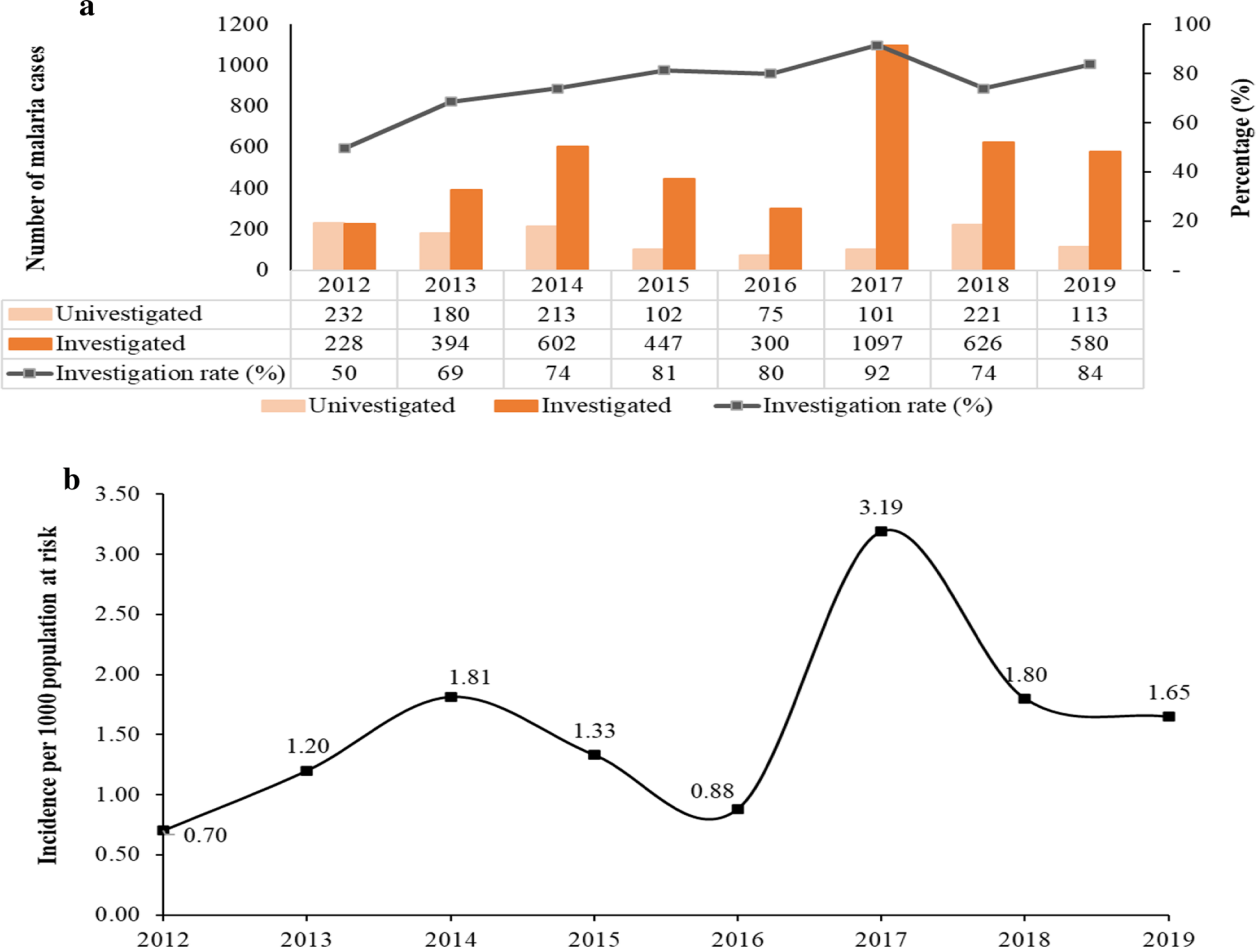
Malaria case numbers and incidence in Eswatini, 2012–2019. **a** Investigated cases, uninvestigated cases, and investigation rate. **b** Incidence per 1000 population at risk of contracting malaria

A total of 5511 patients reported to health facilities between 2012 and 2019 with malaria symptoms. The case investigation rate increased from 50% in 2012 to 84% in 2019, with a record high of 92% in 2017 (Fig. 3a). The number of cases fluctuated in these 8 years, with an upward trend, from a total of 460 cases in 2012 to 693 in 2019 and a peak of 1198 cases in 2017. As the cases increased, so did the malaria incidence per 1000 population at risk, from 0.70 in 2012 to 1.65 in 2019 (Fig. 3b). The highest malaria incidence of 3.19 was recorded in 2017.

**Fig. 4:**
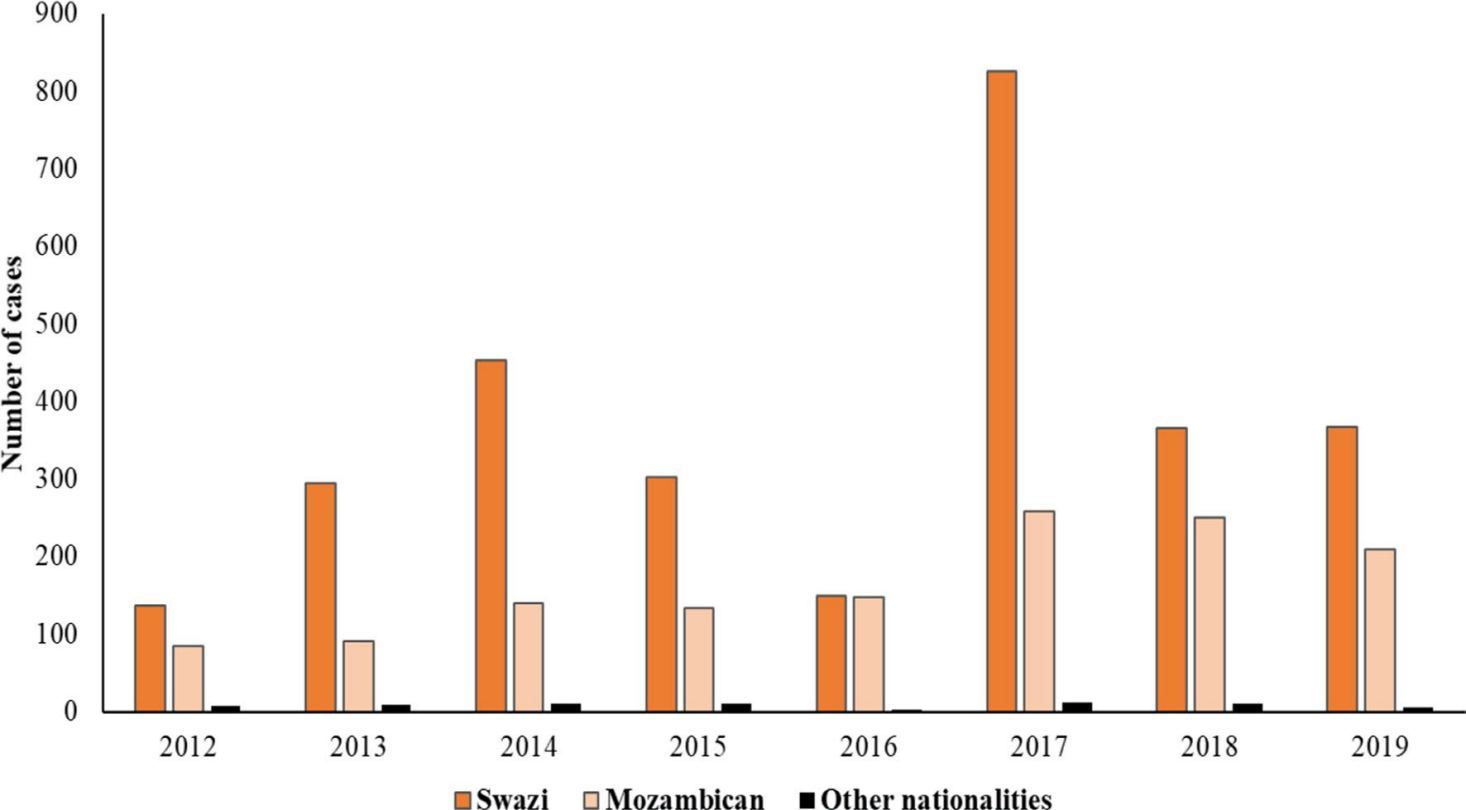
Distribution of malaria cases by nationality in Eswatini, 2012–2019

**Fig. 5:**
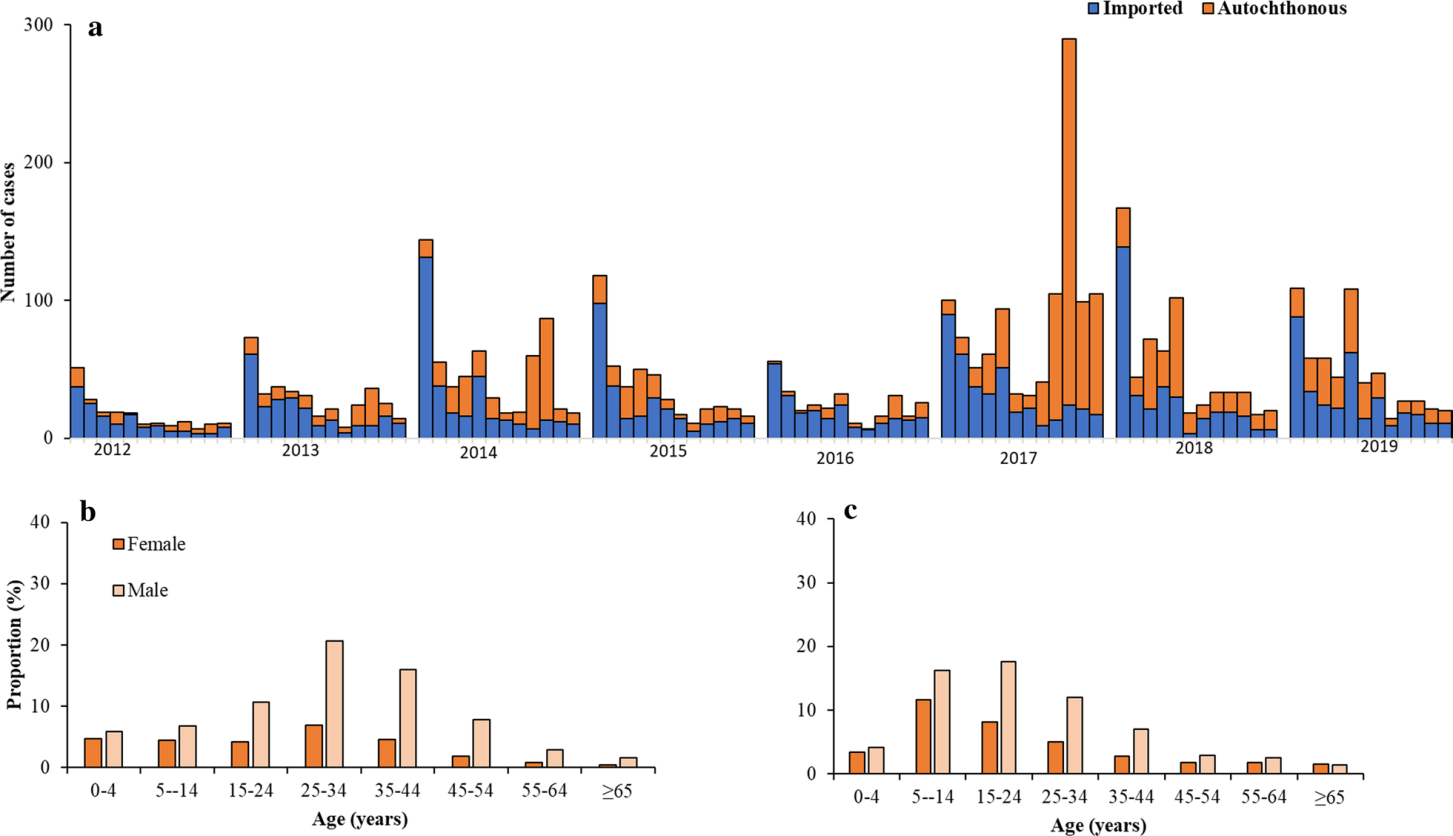
Epidemic curve and demographic features of malaria in Eswatini. **a** Epidemic curve of autochthonous and imported malaria (n = 4173) in Eswatini, 2012–2019. **b** Age of autochthonous male (n = 1194) and female cases (n = 677) in 2012–2019. **c** Age of imported male (n = 1662) and female cases (n = 640) in 2012–2019

**Fig. 6:**
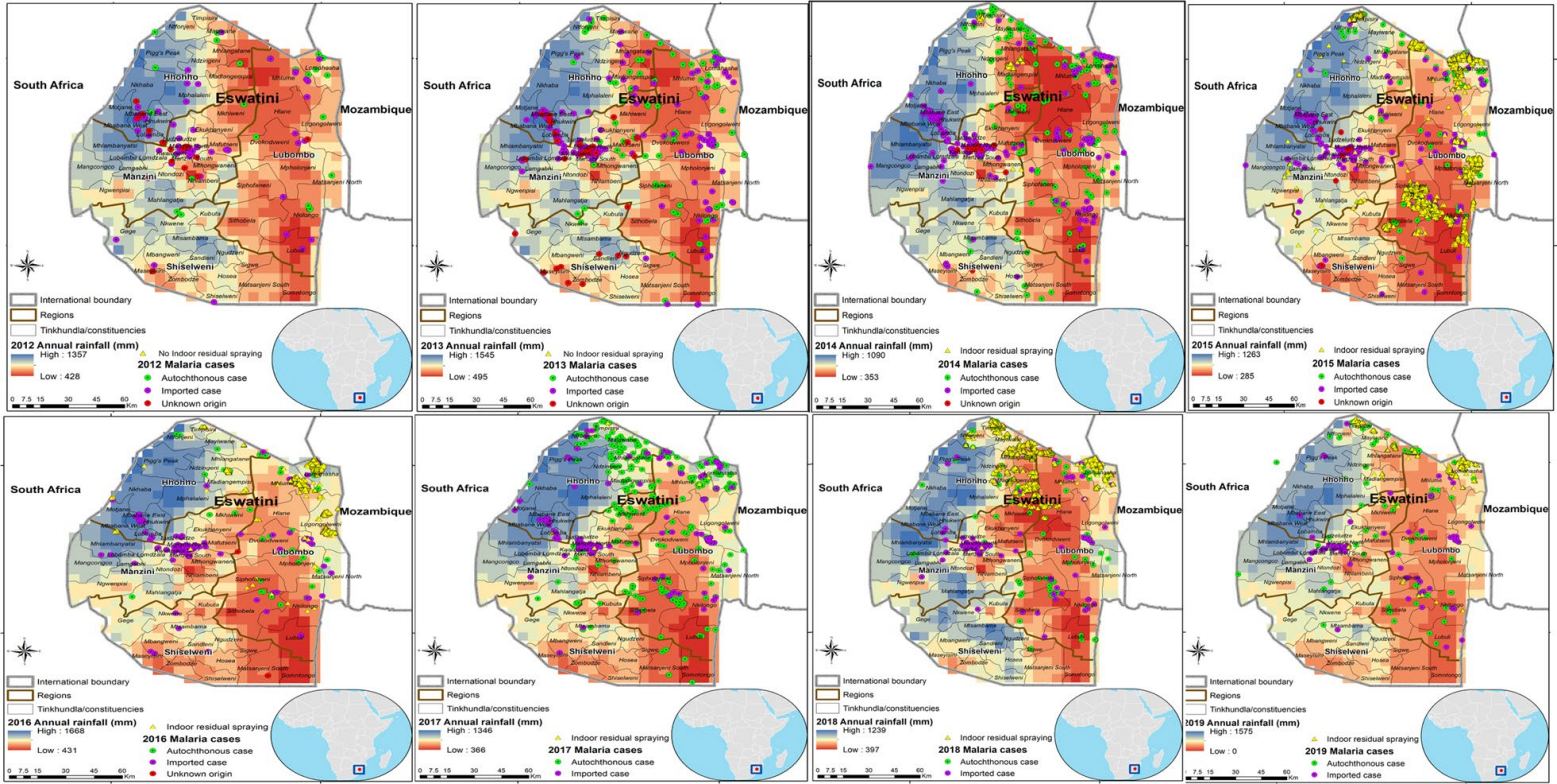
Geographic distribution of malaria cases origin (autochthonous, imported, and unknown), and indoor residua spraying (IRS) in Eswatini, 2012–2019. There was lack of data for 2012 and 2014 even though IRS was done

Malaria remains a major public health problem in Eswatini, with significant transmission occurring in the local communities as shown by the number of autochthonous cases over the years. Most of the investigated cases were Swazi (n = 2895) and Mozambican (n = 1315), with a few from other nationalities (n = 67) (Fig. 4). Whilst in 2012 only 13% (58 out of 460 cases) of the cases were autochthonous, in 2019 over 33% (234 out of 693 cases) were autochthonous (Fig. 5a). Furthermore, as malaria transmission in Eswatini is seasonal, annual data showed a peak in malaria cases in January due to imported rather than autochthonous cases, whilst the local cases peaked later and especially in years with the higher transmission (2014 and 2017), with peak transmission being observed from September to December. In 2017, a year with an exceptionally high number of cases, over 57% of the cases were autochthonous (686 out of 1198 cases). Most autochthonous malaria cases were located along the borders with Mozambique and South Africa and in the Hhohho (middleveld) and Lubombo (lowveld) regions (Fig. 6). Imported malaria cases were found in naturally low malaria risk areas like the central region of Manzini, but also the southern part of the Hhohho region and along the borders with Mozambique and South Africa (Fig. 6). The geographical distribution of cases indicates that local cases occurred in areas supporting transmission (lowveld and lower middleveld), whilst the imported cases to a large extent were seen to occur in the highland areas (highveld and upper middleveld).

There was no IRS data for 2012 and 2013, while in 2014 the data indicates the limited application of IRS and a high number of malaria cases with an increased proportion of autochthonous cases (Fig. 6). In 2015, IRS was very focal, targeting primarily areas that had local transmission in 2014. In 2016, IRS efforts were even more reduced and targeted at the few local transmission hotspots, while in 2017, the year with the highest case incidence rate over the observation period, hardly any IRS was done. In response to the increase in malaria incidence, the areas targeted with IRS in 2018 significantly increased, and focussed especially on Eswatini’s border with South Africa. In 2019, targeted IRS was maintained, keeping cases controlled, with 693 cases reported compared to 847 the previous year (Fig. 6).

Looking at the role of various health facilities in the detection of malaria, most of the cases were diagnosed by RDT in government (86.6%), mission (89.2%), private (87.1%) and company/industry-owned facilities (83.3%), either singly or in combination with microscopy. Mission-owned facilities were more likely to use both RDT and microscopy testing (21.9%) than the other facilities (Table 3).

**Table 3:** Malaria diagnosis methods by ownership of health facility in Eswatini, 2012–2019

Method of diagnosis	Government *n* (%)	Mission/NGO *n* (%)	Private *n* (%)	Company/industry *n* (%)	Unknown *n* (%)
RDT	1568 (76.5)	831 (67.3)	621 (79.4)	113 (65.7)	32 (82.1)
Microscopy	7 (0.3)	15 (1.2)	0	5 (2.9)	0
RDT and Microscopy	206 (10.1)	270 (21.9)	60 (7.7)	32 (18.6)	0
Unknown	268 (13.1)	119 (9.6)	101 (12.9)	22 (12.8)	7 (17.9)
Total	2049	1235	782	172	39

Malaria case surveillance checks if the type of drug prescribed as per national malaria diagnosis and treatment guidelines. The results show that only 58.4% of all uncomplicated cases were treated with AL and single dose primaquine, while only 46.9% of all complicated cases were treated with Artesunate per national guidelines (Table 4).

**Table 4:** Anti-malarial drug prescribed according to malaria severity in Eswatini, 2012–2019

Malaria severity	Drug prescribed						
	AL *n* (%)	AL + Pr *n* (%)	AR *n* (%)	QN *n* (%)	AL + AR *n* (%)	AL + QN *n* (%)	Others *n* (%)
Uncomplicated	747 (96.5)	1221 (99.3)	21 (10.9)	40(35.4)	12 (17.6)	23 (41.8)	25 (100)
Severe	27 (3.5)	8 (0.7)	172 (89.1)	73 (64.6)	56 (82.4)	32 (58.2)	0
Total	774	1229	193	113	68	55	25

*AL* artemether–lumefantrine, *Pr* primaquine, *AR* artesunate, *QN* quinine

## Discussion

Eswatini has made major investments in improving malaria control and surveillance, including significant policy changes enabling the NMP to rapidly respond to cases. Despite all the efforts to make Eswatini malaria-free by 2020, there has been little change over the past decade and the overall elimination strategy has fallen short of its target. Eswatini has managed to keep malaria controlled, with relatively low annual incidence rates compared to its neighbour Mozambique and other E-2020 countries in the region [1, 22]. However, outbreaks could still not be prevented within the case study’s observation period. The reviewed data suggests that higher case numbers are associated with decreased vector control efforts. This is especially well illustrated in 2017 when hardly any structures were sprayed, and local malaria transmission increased rapidly, reaching an unprecedented high over the study observation period. Whilst a surveillance system has been established in the country, the epidemiological case investigation rate is only 84%, with around a fifth of the reported cases remaining uninvestigated. Case classification is based on 3 categories (autochthonous, imported, and unknown), leaving out introduced cases, which are an important marker of local transmission. This must be improved if elimination is to be achieved. Reviewing the NMP databases highlighted significant missing demographic data (GPS coordinates, case origin data) that limited the mapping of malaria cases and IRS coverage. This missing information is pertinent for a country that is aiming for elimination, as all cases need to be identified and mapped for proper and effective deployment of vector control interventions [20].

IRS remains one of the most powerful vector control interventions for reducing/interrupting malaria transmission in terms of its immediate impact. Its use in the last seven decades has played a major role in the elimination of malaria from southern Europe, the Mediterranean region, Russia, large parts of Asia and Latin America, as well as many parts of South Africa [23]. In Eswatini, IRS is supposed to be implemented annually in October, marking one spray cycle before the start of the major local malaria season. This strategy is based on malaria transmission occurring during the warmer and wetter months of November to April. Also, this strategy targets the local cases that seem to peak later in the year as observed in this case study, marking the duration of the transmission season of November–April. The frequency of IRS application depends on, among other factors, the insecticide used and the structure types. Eswatini sprayed DDT in mud structures and pyrethroids in modern/cement structures due to a difference in the residual effect of each insecticide on different wall types. In 2016, the IRS effort was reduced and targeted at the few local transmission hotspots observed in the previous year when IRS was more widely applied. In 2017, hardly any IRS was done. This reduced vector control effort correlated with major outbreaks of local cases in an expanded area of lowveld and lower middleveld regions. The exploration of the data suggests that IRS applications were frequently targeted in areas seen to be persistent malaria hotspots in the previous year. However, this targeted approach might have not considered that the higher coverage with IRS in the previous year prevented most of the cases that would have been seen without intervention. The increase of the IRS efforts in 2018 was associated with reductions in malaria incidence.

The mapped locations receiving IRS from the data provided by the NMP surveillance highlights significant gaps in the strategic deployment of this vector control tool to targeted malaria hotspots in part of the studied period (years). Studies have shown IRS to be an effective strategy for preventing malaria infection and mortality across a range of transmission settings [24–29]. However, low coverage and poor quality of IRS can limit the impact on malaria transmission [29]. Eswatini’s low coverage in 2017 was attributed to challenges in the procurement of insecticide, hence only limited amounts of insecticide (lambda-cyhalothrin) that remained from the previous season were used and targeted at outbreaks rather than prior transmission season hotspots. In 2018, IRS coverage maps show much more spraying. However, the challenges in procurement extended to 2018 and hence whilst there was increased coverage, the timing of IRS was not adhered to and was done late in many targeted regions [14]. In summary, the challenges experienced by the NMP are due to procurement and resource allocation, which led to poor planning and execution of IRS and thus, insufficient coverage. Since IRS is at the core of Eswatini’s vector control strategy, this delay had a major impact on malaria control. To get on track with the elimination effort, the NMP must identify and address the challenges in the implementation of IRS to sustain vector control. Clearly, in Eswatini, logistics is the main challenge in implementing a timely and effective IRS.

Many factors have been shown to contribute to malaria outbreaks in various settings in Eswatini, including rainfall, temperature, population movement, and the lack of sufficient or appropriate control tools or timings of vector control strategies [18]. Control of malaria transmission in border areas, together with the importation of cases, presents a major threat to successfully eliminating malaria in Eswatini. Population movement, especially from the malaria-endemic neighbouring Mozambique, has been previously recorded as an important factor contributing to the persistence of malaria cases in Eswatini [30]. The reviewed data supported these international border movements contributing to malaria cases.

Eswatini can be described as a low-transmission and high-importation case, similar to what was described in a study of Ethiopia, where the local transmission risk was very low, but many cases likely originated from other countries [31]. The high numbers of imported cases that were observed in this Eswatini study during the first few months of the year were likely caused by workers from Mozambique returning to Eswatini in January following the Christmas and New Year holidays [30]. A study conducted in 2016 on travel patterns and demographic characteristics of malaria cases in Eswatini attributed high malaria case importation rates to sugar plantation workers, whose travel patterns are well known between these two countries [30]. Furthermore, the study reported that, since international travellers tend to spend more time away than domestic travellers, they are at a higher risk of getting malaria, especially those travelling to malaria-endemic areas. This length of stay increases the risks of acquiring and returning with parasites. Also, adolescents and employed males were showed to be frequent travellers [30].

Currently, Eswatini’s NMP carries out malaria screening at the Eswatini/Mozambique border, where they do not treat the positive cases but rather refer them to the nearest health facility. The data in this study indicate that outbreaks are due to local transmission, which calls for two different responses: for cases imported to areas where transmission is unlikely, it is more a medical treatment case, so there should be border checks and treatment; whilst for local cases, there needs to be more emphasis on vector control. Elsewhere, it has been previously demonstrated in Eastern Myanmar that early diagnosis and prompt onsite treatment of confirmed cases is effective in achieving malaria elimination [32]. Also, it has been observed in southern Iran that the presence of foreign immigrants could cause malaria outbreaks [33]. Cross-border malaria control initiatives are important in supporting malaria elimination efforts, especially when low-transmission countries share borders with higher-transmission countries. Therefore, there is a need for Eswatini to strengthen its cross-border surveillance, form collaborations with its neighbouring countries, and learn from past lessons such as the cross-border initiative Lubombo Spatial Development Initiative (LSDI) [6]. This initiative represented collaborative efforts between Eswatini, Mozambique and South Africa to reduce each country’s malaria importation risk and achieve elimination. LSDI led to success towards malaria elimination in both South Africa and Eswatini, with IRS as the core intervention [6, 7]. However, the termination of LSDI resulted in an upsurge of malaria cases in these countries, mainly as a result of migration from high-transmission areas to low-transmission ones [6]. The LSDI focus on vector control with IRS further demonstrates the important role of vector control in elimination efforts, and in particular, IRS.

In recent years, Eswatini has engaged in cross-border collaborations with the neighbouring countries of Mozambique and South Africa [14] and regional collaborations via the E-8 initiative [3] as well as partnering with development partners in efforts to tackle cross-border malaria transmission and to augment national efforts towards elimination. The E-8’s mandate has a particular focus on Migrants and Mobile Populations (MMPs) where Eswatini is a recipient of funds through the Initiative to establish malaria border health facilities for Testing, Treating and Tracking (T3). The Mozambique South Africa Swaziland (MOSASWA) cross-border initiative focuses on helping countries to set up mobile clinics along the borders of these three countries [3], however, since Eswatini is a recipient of E-8 funds for the same purpose, the country reprogrammed its budget to focus on IRS, Entomological Surveillance, and Information Education Communication (IEC) [14]. Despite the presence of these mobile clinics along the border, Eswatini still had cases along the border, both autochthonous as well as imported in the study period.

Indeed, community involvement plays an important role in efforts to achieve malaria elimination as the success of interventions, including indoor residual spraying (IRS) and community case management, are effective only if they are accessible, acceptable, and properly used within communities. Many of the challenges to malaria elimination are site-specific and require a more tailored approach to effectively target the remaining malaria foci of transmission and populations at higher risk [34]. Eswatini’s NMP used community engagement platforms, stakeholder meetings, community radio stations, song and dance, roadshows, community drama, as well as home visits to involve communities in information-sharing and collaborative capacity building that sensitized communities on the elimination agenda [35].

Accurate laboratory diagnosis is essential, especially with the adoption of the T3 initiative. False-negative results can lead to untreated malaria and potentially severe consequences, including death. Surveillance systems need to capture true malaria cases for informed interventions. The WHO ‘A Framework for Malaria Elimination’ recommends in the monitoring and evaluation that a percentage of microscopy results be cross-checked by a national reference laboratory for 100% of positive results and 10% of negative results [22]. This study observed that most malaria cases were confirmed using RDT in all health care facilities, while mission/NGO-owned facilities had a higher proportion of cases confirmed by microscope. Even though Eswatini has National Quality Assurance Guidelines for Malaria Diagnosis [34] in place, the NMP has not been routinely implemented, and data was not updated in the ACD database for the samples that were checked for quality assurance. The NMP further stated that it was understaffed and lacked the capacity for routine implementation. Eswatini needs to emphasize the implementation of its guidelines by assessing the epidemiological, operational and financial situation of the malaria programme as recommended by the WHO [22] if it is to attain elimination in the future.

Adherence to the National Malaria Diagnosis and Treatment Guidelines is critical if malaria elimination is to be achieved. Almost all (85%) of the confirmed malaria cases in Eswatini were uncomplicated. However, only a little over a half (58.4%) were treated with AL + primaquine, while only 46.9% of severe malaria cases were treated with artesunate. In contradiction with recommendations in the national diagnosis and treatment guidelines, some cases of uncomplicated malaria were treated with quinine and artesunate, while some patients with severe malaria were treated with AL. Mistreatment of malaria cases could result in worsening of the patient's health status or even death. Non-adherence to national guidelines for malaria treatment has been reported in other African countries such as Uganda [36], Nigeria [36], and Tanzania [37], but none of these countries is at the frontline of malaria elimination, unlike Eswatini. Several factors have been cited for the flouting of national guidelines by clinic staff, including delay in producing laboratory results [36, 38], inadequate supplies of the recommended drugs, and inadequate training of the prescribers [39]. It is therefore crucial for Eswatini to conduct an in-depth evaluation of the possible factors for the non-adherence of national guidelines to generate information to improve case management to achieve malaria elimination.

There are limitations in this study considering this was a retrospective study using secondary data for analysis. Since this data was already entered in the database, there was a possibility of missing data and/or wrong entry in some of the records. Health facility data has the potential for under-reporting malaria cases as a considerable proportion of people may not have presented at the health facilities due to factors such as accessibility. Furthermore, unavailable (missing) data on the mobile population and labour force such as case demographics, reasons for travelling and length of stay when travelled, made it impossible to present data on mobile populations and labour force. Also, other factors may have confounded the observed results, such as the impact of malaria control activities as well as host- and mosquito-related ecological and environmental factors. This study also looked at vector control with IRS; however, for the years 2012 and 2013, there was no IRS data. The NMP explained that the missing/lost information resulted from the Programme modifying its database during the study years.

This case study has programmatic implications. IRS has in the past been successfully proven to work in Eswatini to manage cross-border transmission via the LSDI regional malaria control collaboration [6] and has for over 70 years contributed to eliminating malaria from various countries when integrated with other measures [23]. Integrated vector management (IVM) is the rational decision-making process to maximize the impact of resources allocated for vector control for long-term sustainability [40]. It might be time for Eswatini to consider an integrated approach for malaria control by adding tools such as long-lasting insecticidal nets (LLINs) [41], screening of house entry points [42] and targeted larviciding [43] along with chemoprophylaxis to their malaria control toolbox. Operational research should support such efforts towards IVM [44], which has been demonstrated in other countries including Zambia [45] and Tanzania [46, 47]. In Zambia, the interventions include IRS, LLINs, larviciding and environmental management implemented in eligible urban and rural areas [45]. In Tanzania, integrated control of urban mosquitoes in Dar es Salaam using community sanitation supplemented by larviciding was successful in managing mosquitoes [46, 47].

Furthermore, there is a need to improve entomological surveillance in Eswatini to identify and monitor malaria vectors. Despite the country’s emphasis on vector control, surprisingly little is known about the local vector species and population dynamics, the role of secondary vectors in malaria transmission and the status of insecticide resistance. Monitoring and evaluation indicators for interventions in an elimination programme for vector control calls for independent vector surveys targeting local vectors [19]. This is a challenge Eswatini still faces because its core intervention is IRS and yet there is a lack of crucial ecological data on local malaria vectors, making the emphasis of such intervention lack factual justification. Equally, implementation of resistance management strategies and alternative approaches, including natural-based interventions, will be pivotal for effective IVM and attainment of the objectives of the Stockholm Convention [40]. A review of procedures and challenges at the programme level might help to improve vector control implementation, including routine entomological surveillance in sentinel sites in the different ecological zones. Overall, the review of the malaria control effort over the past 8 years highlights the need to invest in strengthening human resources and infrastructural capacity. These include training and retaining personnel with the necessary skills, establishing laboratories, an insectary, systems for timely procurement and appropriate storage, and adherence to standard operating procedures.

The achievement of malaria elimination requires the involvement of stakeholders in strategic planning and solicitation of funds as well as implementing strategies to achieve the desired goal of malaria elimination. Through Eswatini’s continental partnerships with the African Leaders Malaria Alliance; ALMA [35], and international stakeholders such as WHO-AFRO and Roll Back Malaria (RBM) [35], the country can and must leverage on vast capital and human resource networking. For instance, through ALMA’s scorecard for accountability and action, countries track malaria data to spur action and drive progress towards the goal of ending malaria mortality and morbidity [35]. Furthermore, through a partnership with WHO-AFRO, Eswatini benefits from financial and technical support [14] as well as the opportunity to collaborate with international organizations, such as the International Centre of Insect Physiology and Ecology (*icipe*) [14] which provides technical support to the programme. It is, therefore, important for Eswatini to utilize such partnerships and collaborations to address challenges the challenges that hindered the country from achieving elimination.

## Conclusion

This case study has presented a descriptive analysis of Eswatini’s malaria elimination effort over the past 8 years. Whilst overall malaria incidence rates have remained low, sporadic outbreaks could not be prevented, and they set back Eswatini’s malaria elimination goal of eliminating malaria by 2020. The country needs to review the malaria elimination strategic plan and set a more realistic goal for achieving a malaria-free Eswatini. An integrated vector management approach with a more diverse set of tools and strong community engagement and participation is recommended for higher impact and sustainability.

## Data Availability

Relevant data included in the manuscript.

## Abbreviations

ACD: Active case detection
AL: Artemether–lumefantrine
ALMA: African Leaders Malaria Alliance
DDT: Dichloro-diphenyl-trichloroethane
EPR: Emergency Preparedness and Response
GPS: Global Positioning System
GTS: Global Technical Strategy for Malaria 2016–2030
IDNS: Instant Disease Notification System
IEC: Information Education Communication
IRS: Indoor residual spraying
IVM: Integrated vector management
LLINs: Long-lasting Insecticidal Nets
LSDI: Lubombo Spatial Development Initiative
MOSASWA: Mozambique South Africa Swaziland
NGO: Non-governmental Organization
MMPs: Migrants and Mobile Populations
NMESP: National Malaria Elimination Strategic Plan
NMP: National Malaria Programme
RACD: Reactive case detection
RBM: Roll Back Malaria
RDT: Rapid diagnostic test
SADC: Southern African Development Community
SARA: Service Availability and Readiness Assessment
TTT: Testing, Treating and Tracking
WHO: World Health Organization
WHO-AFRO: World Health Organization Regional Office for Africa
